# The impact of urban green spaces on obesity-related eating behaviours among university students across 31 Chinese cities

**DOI:** 10.7189/jogh.16.04059

**Published:** 2026-02-20

**Authors:** Tingzhong Yang, Sihui Peng, Randall R Cottrell

**Affiliations:** 1 Maternal and Child Healthcare Service Department, Yongkang Women and Children’s Health Hospital, Yongkang, China; 2 Research Center for Digital Health Behavior Theory and Management, Zhejiang University National Health Big Data Institute, Hangzhou, China; 3 Injury Control Research Center, West Virginia University, Morgantown, USA; 4 School of Medicine, Jinan University, Guangzhou, China; 5 Public Health Studies Program, School of Health and Applied Human Sciences, University of North Carolina, Wilmington, USA

## Abstract

**Background:**

Overweight and obesity are well-established risk factors for numerous chronic diseases, but few studies have focused on obesity-related eating behaviours (OEB). Studies suggest that living near green spaces is associated with higher physical activity levels, highlighting the need to examine how these environments specifically influence behaviours related to obesity. Therefore, we aimed to investigate the impact of urban green spaces on OEB among university students across 31 Chinese cities.

**Methods:**

A total of 11 659 students across 31 cities participated in the study. Each respondent completed a standardised questionnaire focused on health behaviour and health in China. We obtained the data on regional characteristics from the National Bureau of Statistics. We used multilevel logistic regression models to examine the associations between urban green space and OEB. Furthermore, we conducted a quantitative analysis to demonstrate the dose-response relationship between the city-level green space and the prevalence of OEB.

**Results:**

Approximately 5.4% of students experienced OEB. According to a multilevel logistic regression analysis, greater availability of green land was significantly associated with a lower likelihood of OEB (odds ratio = 0.44; 95% confidence interval = 0.24, 0.67). There was also a significant inverse dose-response relationship between city-level green land area and the probability of OEB (*β* = −0.2784; *P* < 0.01).

**Conclusions:**

These results reinforce existing literature demonstrating the beneficial role of green spaces in reducing stress and improving mental well-being. Future research should examine variations in student engagement with green spaces in urban environments and explore how natural environment elements can be incorporated into public health strategies across Chinese universities.

Overweight and obesity are well-established risk factors for numerous chronic diseases, including type 2 diabetes, heart disease, hypertension, and certain cancers [1,2]. They also adversely affect mental well-being, with individuals experiencing higher rates of depression, anxiety, and low self-esteem [1]. The broader economic toll is substantial; if current trends continue, global costs are projected to reach USD 3 trillion annually by 2030 [2]. In addition, overweight and obesity reduce workforce productivity through illness, absenteeism, and disability [3], compounding societal burdens.

Urban green spaces have been increasingly recognised for their potential to promote health and well-being, including mitigating overweight and obesity. Numerous studies have examined their impact on physical activity and body mass index outcomes [4–8]. However, the behavioural mechanisms underlying these associations remain underexplored – particularly in terms of obesity-related eating behaviours (OEB), such as emotional eating, stress-induced snacking, and excessive consumption of unhealthy food [9,10].

A limited number of recent empirical studies have begun to address this gap. For example, Wang *et al.* [11] conducted a multi-city study in China showing that perceived access to green space was associated with reduced emotional distress and lower frequency of stress-related eating among young adults. Kondo *et al.* [12] reviewed evidence linking exposure to green space to improved dietary choices, suggesting that psychological restoration in natural environments may reduce impulsive eating. Furthermore, Huang *et al.* [13] demonstrated that street-level greenery influenced emotional responses and indirectly shaped food-related behaviours in urban populations. These findings support the hypothesis that green environments can influence eating behaviour through emotional and cognitive pathways.

To explain this pathway, we draw on a refined interpretation of the stimulus-response-outcome model [14]. In this context, urban green space serves as an environmental stimulus that elicits mental responses, such as reduced stress and improved mood, which, in turn, influence behavioural outcomes – specifically, eating behaviours. This framework supports the hypothesis that exposure to green environments may reduce the likelihood of OEB by enhancing emotional resilience and self-regulation.

Similarly, behavioural susceptibility theory suggests that individuals are more likely to engage in healthy behaviours when their environment facilitates positive affect and social interaction [14,15]. Green spaces offer such settings, encouraging outdoor engagement and reducing time spent in sedentary indoor contexts where unhealthy eating often occurs (*e.g.* watching television, browsing the internet).

Focusing on OEB offers a more proximal and modifiable target for intervention. Unlike overweight and obesity, which reflect cumulative outcomes, eating behaviours are directly shaped by environmental and psychological stimuli and can respond more immediately to changes in context. Green spaces may influence OEB by improving emotional regulation and reducing stress – two key drivers of unhealthy eating patterns.

## METHODS

### Study design and sampling procedure

We followed a cross-sectional, multilevel observational approach, utilising a multi-stage cluster sampling method. In the first stage, we selected 31 universities offering medical programmes from across China. Among these, 18 were specialised medical universities focused primarily on health-related programmes, while 13 were comprehensive universities offering both medical and non-medical courses. In the second stage, we selected academic departments within each university that offered medical or health-related courses. In the third stage, we randomly selected one-third of the courses from each level, averaging two classes per university. In the fourth stage, we surveyed all students enrolled in the selected classes.

### Data collection

We conducted the survey in accordance with accepted implementation guidelines. We administered it during regular class sessions, with an average completion time of 30 minutes. All responses were anonymous. We followed a standardised research protocol across all 31 universities to ensure consistency in questionnaire administration and data collection methods.

### Dependent variable

We defined OEB with three common behaviours associated with high-calorie intake in Chinese culture: consumption of sweet snacks (‘Do you eat sweet snacks?’), overeating leading to excessive caloric intake (‘Do you consume excessive calories?’), and consumption of fatty foods (‘Do you eat fatty foods?’) [14]. The response categories were: ‘Almost never’, ‘Yes, several days’, and ‘Yes, every day’.

The three OEBs we measured share the common characteristic of being high-calorie behaviours that are linked to overweight and obesity [16]. To assess their coherence as a latent construct, we conducted an exploratory factor analysis, which showed that all three items loaded onto a single factor, with factor loadings of 0.69 for sweet snacks, 0.74 for fatty foods, and 0.45 for excessive calories. Exploratory factor analysis indicated that the three items loaded onto a single factor, which explained 42.3% of the total variance. This level of explained variance is considered acceptable in behavioural research, particularly when measuring complex constructs with few indicators [17]. Each behaviour was also significantly associated with obesity-related health outcomes [16]. Furthermore, the study sample comprised university students, and data collection took place in classroom settings during scheduled instructional periods. This controlled environment facilitated strong participant compliance and response accuracy, yielding a high test-retest reliability of 94.8% and a Kappa coefficient of 0.82.

To operationalise OEB as a dependent variable, we defined it as the presence of two behaviours reported as ‘every day’ and one as ‘several days’. We determined this threshold based on established principles for categorising continuous variables [14] and prior empirical applications in similar contexts [16]. To assess the robustness of our classification, we conducted additional analyses using alternative thresholds for the OEB variable. The results remained consistent across these specifications, reinforcing the validity and stability of our categorisation approach. Although prior studies have not uniformly defined OEB in this way, similar composite behavioural indices have been used to capture multi-dimensional eating patterns in obesity research [11], supporting the use of aggregated behavioural indicators in population-based studies.

### Independent variable

We measured the amount of green space in a city by the *per capita* public green land area in a city (ha per 1000 persons) based on data from the Department of Urban Social Economic Survey of the National Bureau of Statistics [18]. Green space refers to areas such as public parks, green spaces within institutions, residential green zones, industrial or production-related greenery, protective vegetation (*e.g.* along river margins), and green landscapes in scenic sites. Notably, *per capita* green land represents the average quantity of green space allocated to residents within a city. Although it functions as a proxy for individual exposure to greenery, this measure reflects city-level provision and does not capture variations in access, distance, or quality of green space across different neighbourhoods. As such, it should be viewed as a preliminary metric for exploring potential associations between urban green environments and OEB.

### Control variables

#### Individual control variables

We included sociodemographic questions, including age, sex, ethnicity, parental occupation, monthly expenses, and major, as confounding variables to further determine the relationships between urban environmental green spaces and OEB.

#### University control variables

We based the university classification on the tiered system defined by the National Ministry of Education in China, categorising institutions as high, middle, or low. Top-tier universities receive significantly more governmental support compared to lower-tier ones and tend to attract students from more advantaged backgrounds and different regions [19].

#### Environmental control variables

The first factor we considered was students’ urban or rural home background. Students from rural areas, due to their poorer living conditions, were expected to exhibit lower levels of overweight and obesity than their urban counterparts [20]. Second, previous research has shown that overweight and obesity tend to be higher among students from economically disadvantaged provinces [20,21]. Therefore, we included two indicators of regional economic development: the gross domestic product of the university city and the average wage of employed individuals and workers (in CNY, Yuan) [18].

### Modelling strategy

We first entered the data in Microsoft Excel, version 2601 (Microsoft Corp., Redmond, Washington, USA). We used descriptive statistics to estimate the prevalence of OEB. We employed logistic regression models to examine the relationships between the outcome variable and each independent variable. We conducted crude and adjusted analyses, with the latter implemented through multilevel regression models. Controlled variables included demographic characteristics, monthly expenses, average wage, university type, and the gross domestic product of the university’s city (**Table 1**). All statistical procedures incorporated weighting, as detailed by Yang *et al.* [22].

**Table 1:** Sample characteristics and prevalence of OEB

	n (%)	Prevalence, %	OR (95% CI)	*P*-value
**Age in years**				
<18	1873 (20.2)	5.6	ref	
19	2523 (20.3)	6.3	1.14 (0.57, 2.29)	0.712
20	2993 (22.5)	5.4	0.97 (0.65, 1.46)	0.883
21	2370 (18.8)	4.7	0.83 (0.43, 1.63)	0.584
≥22	1900 (18.2)	4.4	0.77 (0.36, 1.65)	0.501
**Gender**				
Male	3722 (33.2)	5.7	ref	
Female	7937 (66.8)	5.1	0.88 (0.68, 1.13)	0.324
**Ethnicity**				
Han	10 713 (86.0)	4.3	ref	
Minority	946 (14.0)	11.1	2.78 (1.48, 5.22)	0.001
**Father’s education level**				
Elementary school or less	1980 (35.0)	7.5	ref	
Junior high school	4511 (36.6)	4.2	0.54 (0.29, 1.01)	0.053
High school	2741 (20.8)	4.5	0.57 (0.25, 1.32)	0.185
Junior college	1294 (11.3)	5.2	0.68 (0.32, 1.44)	0.314
College	1133 (6.2)	6.2	0.82 (0.46, 1.49)	0.508
**Mother’s education level**				
Elementary school or less	3201 (33.1)	5.7	ref	
Junior high school	4347 (34.4)	4.5	0.78 (0.48, 1.28)	0.321
High school	2250 (16.9)	4.8	0.84 (0.45, 1.57)	0.584
Junior college	1114 (9.7)	6.2	1.09 (0.56, 2.11)	0.799
College	747 (5.9)	7.6	1.35 (0.79, 2.30)	0.271
**Father’s occupation**				
Managers	890 (7.2)	7.4	ref	
Professionals	767 (6.7)	7.4	0.97 (0.54, 1.82)	0.922
Business and service	1913 (14.6)	6.1	0.81 (0.54, 1.21)	0.306
Technical worker	1785 (15.0)	3.8	0.49 (0.32, 0.75)	0.001
Operations	3359 (29.4)	4.6	0.61 (0.31, 1.20)	0.152
Retired	150 (1.4)	4.6	1.28 (0.39, 4.23)	0.685
No employment	515 (5.0)	5.6	0.74 (0.30, 1.86)	0.518
Others	2280 (20.7)	5.1	0.68 (0.38, 1.20)	0.189
**Mother’s occupation**				
Managers	511 (4.6)	9.1	ref	
Professionals	782 (6.6)	10.5	1.17 (0.74, 1.85)	0.502
Business and service	2017 (15.8)	6.1	0.66 (0.39, 1.10)	0.116
Technical worker	823 (6.8)	2.5	0.26 (0.11, 0.60)	0.002
Operations	2781 (24.5)	4.5	0.46 (0.26, 0.85)	0.010
Retired	290 (2.2)	6.3	0.67 (0.36, 1.26)	0.210
No employment	2181 (18.8)	4.4	0.47 (0.28, 0.80)	0.005
Others	2274 (20.5)	4.8	0.50 (0.31, 0.81)	0.005
**Monthly expenses in CNY**				
<1000	3998 (33.7)	3.1	ref	
1000–1499	5364 (48.6)	5.1	1.65 (1.30, 2.11)	<0.001
1500 and over	2297 (24.9)	8.6	2.89 (2.05, 4.08)	<0.001
**Major**				
Public health	2589 (24.6)	5.7	ref	
Clinic Medicine	6402 (59.6)	5.3	0.93 (0.60, 1.43)	0.743
Nursing	1067 (6.5)	6.0	1.05 (0.68, 1.63)	0.827
Others	1601 (9.2)	3.8	0.65 (0.31, 1.37)	0.256
**University type**				
Lower level	3276 (17.0)	3.3	ref	
Middle level	7481 (68.1)	6.0	1.91 (0.79, 3.74)	0.103
High level	902 (14.8)	4.3	1.35 (0.78, 2.32)	0.280
**University city GDP in CNY**				
<8000	6084 (58.6)	6.3	ref	
8000–11 999	3445 (25.7)	3.2	0.59 (0.41, 0.91)	0.009
12 000 and over	2130 (15.7)	4.9	0.75 (0.46, 1.23)	0.252
**Average wage of employed individuals and workers in CNY**				
<5000	2643 (25.8)	3.2	ref	
5000–7999	7331 (53.1)	4.9	1.54 (1.01, 2.35)	0.045
≥8000	1685 (21.2)	8.4	2.90 (1.52, 5.51)	0.001
***Per capita* green land area, in ha per 1000 persons**				
<30	3171 (26.9)	7.7	ref	
30–39	5956 (64.3)	4.8	0.60 (0.30, 1.18)	0.144
≥40	2532 (8.7)	1.9	0.23 (0.08, 0.66)	0.006

CI – confidence interval, GDP – gross domestic product, OEB – obesity-related eating behaviour, OR – odds ratio, ref – reference

To assess multicollinearity among the independent variables, we calculated variance inflation factors (VIFs) using standard linear regression techniques. We entered each predictor into a full model and computed VIFs to quantify the extent to which the variance of each estimated coefficient was inflated due to correlations with other predictors. We considered VIF < 5 acceptable, indicating no serious multicollinearity concerns [23].

We developed a series of models to identify predictors of OEB. The analysis began with a null model – a two-level structure (individual and university city) with random intercepts – that included only a constant term, serving to assess baseline variation at the city level. Building on this, three models were constructed. Model 1 included individual-level variables: ethnicity, mother’s occupation, monthly expenses, and green land area. Model 2 combined both individual- and regional-level variables, including ethnicity, mother’s occupation, monthly expenses, city-level average wage, and green land area.

In multilevel models, interpreting both fixed and random effect parameters is essential for understanding the model structure and results. Fixed effects represent the average influence of independent variables on the dependent variable across all units. In contrast, random intercepts capture baseline variation in the outcome variable across groups.

We evaluated model fit using multiple information criteria, including the −2 log likelihood, Akaike, Bayesian, and Hannan-Quinn information criteria. Lower values of these indices indicate better model fit, and we used them to compare both nested and non-nested models. To assess potential overdispersion in the multilevel logistic regression models, we examined the Pearson chi-square statistic (χ^2^) divided by its degrees of freedom (df). A χ^2^/df substantially greater than one is commonly interpreted as evidence of overdispersion, suggesting that the observed variability exceeds expectations under the assumed binomial distribution [24]. Conversely, a χ^2^/df < 3 is generally considered indicative of acceptable model fit and reasonable residual variance [25].

In addition, we conducted a quantitative analysis to demonstrate the dose-response relationship between city-level green land area and the prevalence of OEB across cities using regression coefficients (*β*) and scatterplots.

We used SAS, version 9.3 (SAS Institute Inc., Cary, North Carolina, USA) for all statistical analyses.

## RESULTS

We initially considered 11 802 individuals for inclusion. Of these, 11 783 students from 31 universities participated. After removing responses that were incomplete or logically inconsistent, the final sample comprised 11 659 students. Notably, we administered the survey in classrooms during scheduled class sessions, resulting in a high response rate given the low student absenteeism rate.

Among the study participants, 20.2% were <18 years old, 42.8% 18–21 years old, and 37.0% ≥21 years old. Additionally, 68.8% were females, and 86.0% identified as Han Chinese (**Table 1**).

The VIF analysis revealed that most of the predictors had VIF <5, indicating no evidence of severe multicollinearity. Most variables exhibited VIFs between 1.2 and 5.0, which are generally considered acceptable. Only the variable representing maternal occupation as ‘Operations’ had a slightly elevated VIF of 5.02, suggesting moderate multicollinearity. Overall, these results support the reliability of the regression estimates and indicate that multicollinearity is not a major concern in the current model specification.

Model fit statistics indicated progressive improvement from the null model to the individual-level and full models. The −2 log likelihood decreased from 161 089.7 (null model) to 155 104.9 (full model), with corresponding reductions in Akaike, Bayesian, and Hannan-Quinn information criteria. These results suggest that including individual-level covariates substantially improved model fit. Conditional fit statistics further supported the improved performance of the individual-level and full models, with −2 log likelihood values converging around 154 940. Notably, χ^2^/df decreased across models: 1.21 for the null model, 0.98 for the individual-level model, and 0.95 for the full model. These values indicate a good model fit and suggest that the overall residual variation falls within an acceptable range.

Among the participants, OEB prevalence was 5.4% (95% confidence interval (CI) = 4.7, 5.9). According to multilevel logistic regression analysis (**Table 2**), greater availability of green land was significantly associated with a lower likelihood of OEB (adjusted odds ratio (aOR) = 0.44; 95% CI = 0.24, 0.67). Additionally, higher monthly expenses, specifically CNY 1000–1499 (aOR = 1.43; 95% CI = 1.26, 1.63) and CNY 1500 and over (aOR = 2.14; 95% CI = 1.53, 2.99), were positively associated with OEB in the individual and regional combined model. Similarly, higher average wages of CNY 5000–7999 (aOR = 1.68; 95% CI = 1.17, 2.40) and CNY 8000 and over (aOR = 2.19; 95% CI = 1.50, 3.19) were also positively associated with OEB.

**Table 2:** Individual and regional influences on OEB

	Individual model		Individual and regional model		
	**aOR (95% CI)**	***P*-value**	**aOR (95% CI)**	***P*-value**
**Ethnicity**				
Han	ref		ref	
Minority	2.98 (1.44, 6.16)	0.003	2.36 (1.49, 3.84)	<0.001
**Mother’s occupation**				
Managers	ref		ref	
Professionals	1.22 (0.69, 2.14)	0.491	1.23 (0.71, 2.13)	0.460
Business and service	0.77 (0.46, 1.29)	0.320	0.74 (0.45, 1.22)	0.237
Technical worker	0.32 (0.14, 0.74)	0.007	0.30 (0.13, 0.68)	0.004
Operations	0.52 (0.31, 0.89)	0.015	0.46 (0.28, 0.77)	0.002
Retired	0.74 (0.41, 1.34)	0.319	0.76 (0.45, 1.30)	0.311
No employ	0.57 (0.34, 0.98)	0.037	0.54 (0.33, 0.89)	0.015
Others	0.52 (0.29, 0.90)	0.024	0.46 (0.26, 0.80)	0.006
**Monthly expenses in CNY**				
<1000	ref		ref	
1000–1499	1.57 (1.33, 1.85)	<0.001	1.43 (1.26, 1.63)	<0.001
1500 and over	2.40 (1.67, 3.47)	<0.001	2.14 (1.53, 2.99)	<0.001
**Average wage of employed individuals and workers in CNY**				
<5000			ref	
5000–7999			1.68 (1.17, 2.40)	0.005
8000 and over			2.19 (1.50, 3.19)	<0.001
***Per capita* green land area in ha per 1000 persons**				
<30	ref		ref	
30–39	0.85 (0.61, 1.23)	0.364	0.84 (0.61, 1.24)	0.335
≥40	0.41 (0.24, 0.67)	<0.001	0.44 (0.24, 0.67)	0.002
Fixed parameters*	4.86	<0.001	3.39	<0.001
Random parameters between cities*	3.41	<0.001	3.26	<0.001

aOR – adjusted odds ratio, CI – confidence interval, OEB – obesity-related eating behaviour, ref – reference

*Null model aOR was 5.02 (*P* < 0.01) for fixed parameters, and 3.48 (*P* < 0.01) for random parameters between cities.

**Figure 1:**
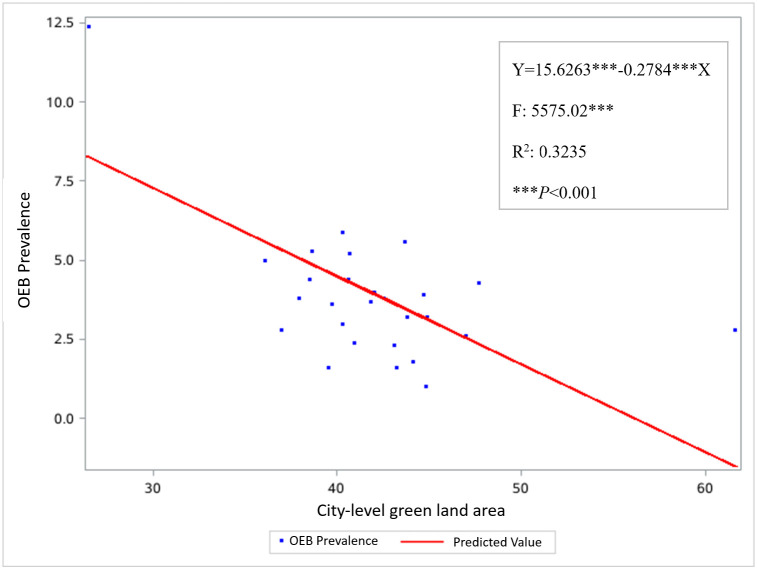
Scatter plot of the association between the city-level green land area and OEB prevalence.

The analysis revealed a significant inverse dose-response relationship between city-level green land area and the probability of OEB (β = −0.2784; *P* < 0.001) (**Figure 1**).

## DISCUSSION

We found that greater access to green land is associated with lower odds of OEB, suggesting a potential relationship between urban green space availability and healthier eating behaviours. A dose-response relationship between city-level green land area and OEB prevalence further supports the categorical findings, aligning with prior research [9,10].

Green spaces are known to reduce stress and enhance emotional well-being [7], which may be linked to a lower likelihood of emotional eating. Exposure to natural environments has been associated with improved emotional regulation and reduced stress-related behaviours, including overeating [26,27].

Access to green space has also been associated with increased outdoor physical activity, which may contribute to more structured routines and healthier dietary patterns. According to the behavioural susceptibility theory [14,15], increased outdoor activity may reduce time spent in sedentary indoor settings – where OEB often occurs – potentially contributing to the observed negative association.

Moreover, greener neighbourhoods tend to exhibit higher social capital and environmental quality, both of which have been linked to healthier eating [28]. Pleasant, safe green areas may also enhance social cohesion, which could, in turn, support healthier lifestyle choices.

Overall, these findings underscore the relevance of urban planning and public health policy in addressing disparities in access to green environments. In rapidly urbanising contexts, integrating green infrastructure into residential and university settings offers a potentially scalable approach to support healthier lifestyles. Such environments may encourage physical activity and reduce sedentary behaviour, which are associated with lower OEB prevalence. By fostering outdoor engagement and well-being, policymakers may help mitigate the burden of lifestyle-related diseases and promote community health. Thus, equitable access to urban green spaces should be considered an important component of public health strategies that support healthy dietary behaviours and long-term well-being.

According to the exposure-resources theory, individuals’ ability to meet their needs depends on the resources available to them [14,29]. When faced with more negative exposures and fewer resources, their capacity to cope diminishes, increasing the risk of poor health outcomes. Socioeconomic status (SES), including education, occupation, and income, is a key resource shaping health trajectories. We found that higher individual SES, occupational standing, monthly expenses, and regional economic status (as measured by city-level average wage) were positively associated with OEB. Several factors may explain this. Firstly, students with greater financial means often have easier access to calorie-dense, high-fat, and sugary foods, such as restaurant meals and snacks. Additionally, affluence may be linked to lifestyle patterns involving frequent social dining or late-night eating, especially in urban university environments. Lastly, in some urban cultures, high consumption of energy-rich foods is normalised as part of a ‘modern’ lifestyle, which may help explain the higher prevalence of OEB among wealthier students.

We found that average wages were positively associated with OEB. Although higher SES is often linked to healthier lifestyles, these findings challenge that assumption by revealing a positive correlation between SES and OEB among university students. Wealthier individuals typically have greater financial resources, allowing access to a wider variety of foods, including energy-dense fast food, desserts, and convenience items [21,30,31]. In Chinese culture, interpersonal relationships are highly valued, and social bonding is often achieved through shared meals [14]. Affluent students may be more likely to engage in high-calorie, high-fat social eating activities as part of their social routines [14,31]. Moreover, in fast-paced urban university environments, elevated stress levels may lead high-SES students to use food as a coping mechanism, contributing to emotional eating patterns [32]. Future research should explore these cultural and contextual mechanisms to better understand how income and social environment influence eating behaviours across different populations.

### Strengths and limitations

A key strength of this study lies in our focus on a predominantly young student population – a group known to experience elevated levels of OEB, overweight, and other health concerns. Although we did not cover all regions, we included university students from diverse social backgrounds and a wide range of institutions across China, enhancing the sample's representativeness.

However, several limitations should be acknowledged. Firstly, the cross-sectional nature of the study precludes causal inference regarding the relationship between green space exposure and OEB. Future research should adopt longitudinal or experimental designs involving students of varying ages, socioeconomic backgrounds, and university settings to clarify temporal dynamics and underlying mechanisms. Secondly, all exposure and outcome variables were self-reported and collected concurrently, which may introduce recall bias and limit the ability to establish temporal relationships. Additionally, we did not measure factors such as dietary knowledge, food environment, and psychological stress, but they may have influenced the observed associations. Furthermore, participants were exclusively students majoring in health-related fields, which may limit the generalisability of findings to the broader population of Chinese college students and young adults. Lastly, we relied on city-level green space indicators (*i.e.* *per capita* green land) as proxies for individual exposure. While this metric reflects overall green space provision within a city, it does not account for individual-level differences in access, proximity, or quality. Moreover, people typically interact with multiple green spaces across different contexts, and the lack of detailed personal exposure data remains a challenge.

To address these limitations, future studies should incorporate objective measures – such as geographic information system-based accessibility indices and behavioural outcomes, such as dietary logs or biomarkers – to strengthen causal inference and validate self-reported data. Additionally, research should move beyond documenting associations to generate evidence that directly informs the planning, design, and implementation of green spaces. This will support a deeper understanding of which green-space features are most beneficial for specific population groups and how such insights can be translated into targeted interventions and policy frameworks for OEB prevention.

## CONCLUSIONS

We provide preliminary evidence that urban green spaces are associated with lower levels of OEB among college students. Given the rising prevalence of overweight and obesity in this population, prioritising OEB prevention within broader public health strategies in China is both timely and necessary. Elevated OEB levels among students may reflect broader trends in younger demographics, highlighting the need for targeted, context-specific interventions. Beyond identifying statistical associations, our findings carry translational significance for urban planning and health promotion. We recommend integrating green-space design into university campuses and adjacent urban areas to foster healthier behavioural environments. Planning efforts should emphasise accessibility, safety, and usability to maximise engagement and potential health benefits. To strengthen causal inference and deepen understanding of underlying mechanisms, future research should adopt longitudinal or experimental designs. Geographic information system-based indices of green space accessibility and behavioural outcomes, such as dietary logs or biomarkers, will be critical for validating self-reported data and clarifying temporal relationships. Research should move beyond documenting associations to generate actionable insights that inform the planning, design, and implementation of green spaces [33,34]. In university city contexts, this approach can help identify which green-space features are most effective for specific population groups and guide the development of targeted public health strategies to reduce OEB.
